# Evaluation of a 25-Year-Program for the Control of Schistosomiasis Mansoni in an Endemic Area in Brazil

**DOI:** 10.1371/journal.pntd.0000990

**Published:** 2011-03-15

**Authors:** Ana K. Sarvel, Áureo A. Oliveira, Alexandre R. Silva, Anna C. L. Lima, Naftale Katz

**Affiliations:** 1 Laboratory of Schistosomiasis, Research Center René Rachou/FIOCRUZ, Belo Horizonte, Minas Gerais, Brazil; 2 Federal University of Ouro Preto (UFOP), Ouro Preto, Minas Gerais, Brazil; 3 Federal University of Minas Gerais (UFMG), Belo Horizonte, Minas Gerais, Brazil; University of California San Francisco, United States of America

## Abstract

**Background:**

Various studies showed that chemotherapy can control schistosomiasis morbidity, but association of measures (water supply, sewage disposal and increase of socioeconomic conditions) is necessary for transmission control.

**Methodology/Principal Findings:**

A survey dealing with socioeconomic conditions, snail survey, contact with natural waters, and clinical and stool examinations was undertaken at an endemic area in the State of Minas Gerais, Brazil. The methodology used was the same for both evaluations (1981 and 2005). Four hundred and seventy-five out of 1,474 individuals studied in 1981 could be contacted. From these, 358 were submitted to stool examination, and 231 of them were clinically examined. Patients eliminating *S. mansoni* eggs in their stools were treated. The results showed that the prevalence rate in Comercinho, a municipality of the State of Minas Gerais, Brazil, was substantially reduced to 70.4% and 1.7% in 1981 and 2005, respectively, as well as the frequency of the hepatosplenic form (7% to 1.3%) after five treatments effectuated between 1981 and 1992. No other new case of this form was detected from 1981 onwards. Another important aspect to be considered was the improvement of people's living standard that occurred in the region after more than two decades' efforts (better housing, professional skill and adequate basic sanitation).

**Conclusion/Significance:**

The control of morbidity and very significant decrease of schistosomiasis transmission in an area until then considered as hyperendemic was possible by means of association of successive specific treatments of the local population, together with the construction of privies, water supply in the houses and improvement of socioeconomic conditions.

## Author Summary

A clinical-epidemiological reevaluation on schistosomiasis mansoni was performed in 2005, in the urban area of a little town, Comercinho, MG, specifically focusing on the inhabitants of the same area in 1981, when a first survey and treatment with oxamniquine were carried out. The surveys included: identification of the intermediary host, census, mapping of the city, socioeconomic survey, stool examination, clinical examination, research dealing with contact with natural waters, and treatment of the positive cases. From a population of 1,474 people studied in 1981, 358 were submitted to stool examination, and 231 were clinically examined. From 1981 to 1992 five specific treatments were performed with oxamniquine and the last one with praziquantel. The results obtained were compared and demonstrated that the prevalence in Comercinho decreased significantly (70.4% to 1.7%), as well as the hepatosplenic form (7% to 1.3%) in 1981 and 2005, respectively. Significant improvement in the life quality (improvement in the housing, professional qualification and basic sanitation) were observed and must be considered important for the schistosomiasis control.

## Introduction

Schistosomiasis is a social disease, found at poor rural regions and periphery of cities, with a precarious socioeconomic development, where the inhabitants have frequent contact with contaminated waters, as well no available of adequate sewerage system.

WHO considers schistosomiasis as the second only to malaria in socioeconomic importance worldwide, and the third more frequent parasitic disease Public Health importance. [1] The main necessary step recommended for reduction of schistosomiasis morbidity is the treatment of individuals living in endemic areas [2]. The program for national control of schistosomiasis was launched in Brazil in 1975, by SUCAM (“Superintendência de Campanhas de Saúde Pública”) – Ministry of Public Health, by means of the “Programa Especial de Controle da Esquistossomose (PECE)” (Special Program for Schistosomiasis Control), which directed its activities for the chemotherapeutic treatment with oxamniquine in large scale (more than 13 million people were treated). As molluscicide, niclosamide was used, but in lower scale and irregular manner. Sanitation, safe water supply and health education were also measures adopted, but with less frequency [3].

Various early studies demonstrated that improvement of the sanitary conditions and treatment of positive patients contribute to reduce morbidity and prevalence of the disease [4]–[11]. A study carried out in Comercinho/MG, Brazil, in 1974, clearly shows these facts. In that year, a staff from the Laboratory of Schistosomiasis, Research Center René Rachou/FIOCRUZ, under the leadership of one of the authors (NK), performed the first survey on schistosomiasis mansoni (prevalence of 70.4%). Census of the population, mapping of the town, clinical and stool examinations of the patients infected with *S. mansoni* were performed. However, the treatment of the local population could not be administered as it was intended, due to the appearance of lethal cases in Brazil with the use of hycanthone, the antischistosomal drug of choice at that occasion. After the discovery of a novel drug – oxamniquine – the researchers came back to Comercinho in 1981. On that year, besides the above mentioned measures, other ones were taken, such as: identification of the intermediate host, socioeconomic survey, research on contact with natural waters, clinical examinations of the population, and intradermal reaction for this group, besides specific treatment of infected patients [12]. In 1984 and 1986, the individuals that presented *S. mansoni* eggs in the feces, detected by means of examination of the local residents performed in the preceding year, were once more treated. In 1988 a new re-evaluation was undertaken according to the same methodology [13]. In 1992, Rocha and Katz [14] re-examined the conditions of the area after five treatments with oxamniquine (from 1981 to 1991). From that date onwards the Prefecture of the town was in charge of the program for the control of schistosomiasis, and the treatments continued to be administered at the local Public Health Center by local physicians and technicians (horizontalization of the Program for Schistosomiasis Control). In 2005, a new clinical-epidemiological survey of the population living in the area in 1981 was carried out, focused on the following priorities: identification of the intermediate host; parasitological, clinical and socioeconomic evaluations of the population and evolution of contact with natural waters.

In the present paper we compare the results of the last evaluation (2005) with data related to the inhabitants of the region in 1981, when people were treated with antischistosomal drug for the first time.

## Methods

### Characterization of the study area

Comercinho is a little town located at the Northeast of the State of Minas Gerais, macro-region of Jequitinhonha, Brazil, at a distance of 714 Km from the capital of the state. In 2005, the population was estimated in 10.181 inhabitants and 3.340 of them were living in the urban area, where this study was done. The urban center has three public buildings pertaining to the Prefecture (1 for odontological attendance, the other ones for the “Programa de Saúde da Família (PSF)” – Program for Family Health – and for the “Programa de Controle da Esquistossomose - PCE” – Program for the Control of Schistosomiasis. COPASA (“Companhia de Saneamento de Minas Gerais”) is responsible for the water supply and sewerage system. The household waste is daily collected in all the urban area, and the solid residues are deposited in a landfill situated 2 Km far from the urban center.

### Ethical Committee for Human Research

The patients were informed about this new study, and a signed written informed consent was obtained from all patients (including from parents/guardians for all the 7–14-year old children) before admission to the study. This study was approved by the Ethical Committee for Human Research of the Research Center René Rachou/FIOCRUZ (02/2006-CEPSH/CPqRR), and by the Ethical Committee for Human Research of the Santa Casa Hospital, in Belo Horizonte/MG (Statement n° 016/2006).

### The studied population

The inhabitants that participated in the study performed in 1981, and were still living in the area, were interviewed by the technicians of the “Programa de Saúde da Família (PSF)”. They answered a socioeconomic questionnaire, and they were also invited to be submitted to clinical and stool examinations.

### Identification of the intermediate host

Collection of snails was performed within the urban area (Sapê and Areia brooks). The snails were sent to the Mollusc Room at the Research Center René Rachou/FIOCRUZ in order to be identified and evaluated in relation to *S. mansoni* infection, by means of light exposure and by crushing between two glass plates.

### Socioeconomic survey

The staff of the “Programa de Saúde da Família (PSF)” visited the housings of the participants in the study performed in 1981, and the interview was held with the owner or user of the housing. The socioeconomic survey considered the following items: a) insertion of the family head into the productive system; b) individual occupation; c) working place; d) place of birth; e) type of housing; f) source of water supply. These topics were assessed according to the same parameters described by Costa in 1983 [12].

### Stool examination

The patients received a recipient to collect the feces, identified with the same plot number attributed to them in 1981. A stool sample was collected for preparation of two slides by the Kato-Katz method [15]. The eggs were counted, and an arithmetic average of the number of eggs per gram of feces was considered as an individual result. In order to know the number of eggs per gram of feces (epg) in the community the geometric average was used. The positive patients (eliminating *S. mansoni* eggs in the feces) received chemotherapeutic treatment for schistosomiasis and/or other helminthiases. Seven hundred fifty-nine school children (7 to 14-year-old) were examined for evaluation of the current situation related to schistosomiasis.

### Clinical examination

Clinical examination was performed by means of anamnesis and abdominal palpation. The clinical classification adopted was: type I (intestinal), type II (hepatointestinal) or type III (hepatosplenic) [16].

### Contact with natural waters

The patients clinically examined answered a questionnaire on contact with natural waters, for evaluation of the frequency and reason for contact. The data were grouped as follows: washing clothes, fetching water, bathing, leisure (swimming and/or fishing), professional activities (watering garden, removing sand and crossing the stream).

### Statistical methods

The chi-square test was used for comparison of frequency distribution (1981–2005). The test was performed with 95% confidence, using the program SPSS version 11.5.

## Results

### Identification of the intermediate host

One hundred and sixteen out of the 181 captured snails were found to be alive, and were identified as *Biomphalaria glabrata*. None of the alive snails examined were infected with *Schistosoma mansoni*.

### Comparison between the variables of the population studied in 1981 and re-evaluated in 2005

**Figure 1:**
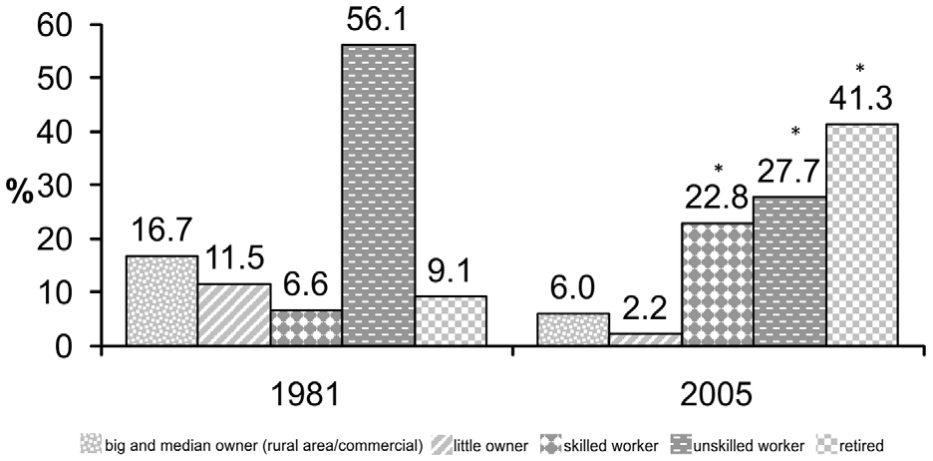
Data on the activities of the family heads in Comercinho/MG, Brazil (1981–2005). The figure 1 shows the proportion related to the heads of the households considered as skilled workers, which increased throughout the mentioned years. * p-value<0.001. The figure 1 shows the proportion related to the heads of the households considered as skilled workers, which increased throughout the mentioned years.

In 2005, 475 out of 1.474 individuals that have participated in the study carried out in 1981 could be contacted. Stool and clinical examinations were performed in 1.329 and 836, and in 358 and 231 individuals, in 1981 and 2005, respectively. Table 1 shows comparison of the socioeconomic survey obtained in 1981 and 2005. As can be seen in Table 1, significant improvements were attained, such as substantial increase in the number of housings with safe water supply provided by the Public Service (from 33.7% in 1981 to 96% in 2005). Waste disposal using cess-pits or flush toilets was increased from 71.7% to 97.6%, provided by the population. In 1981, only 34.2% of the housings were classified as type A (considered of better quality), and 97.6% in 2005. The proportion related to the heads of the households considered as skilled workers showed also a significant increase (6.6% to 22.8%) (Figure 1).

**Table 1:** Comparative data of the socioeconomic survey carried out in Comercinho/MG, Brazil (1981–2005).

VARIABLES		1981(N)%	2005(N)%
**1. Number of participants**		**(1474) 100.0**	**(358) 100.0**
**2. Local of birth**			
Urban area		(768) 52.0	(177) 49.5
Other		(706) 48.0	(179) 50.5
	**Total**	**(1474) 100.0**	**(358) 100.0**
**3. Water supply** *			
Public service		(94) 33.7	(237) 96.0
Other		(185) 66.3	(10) 4.0
	**Total**	**(279) 100.0**	**(247) 100.0**
**4. Water disposal** *			
Cess-pit/Flush toilet		(200) 71.7	(241) 97.6
Other		(79) 28.3	(6) 2.4
	**Total**	**(279) 100.0**	**(247) 100.0**
**5. Type of the housings** *			
Type A		(94) 34.2	(241) 97.6
Type B		(91) 33.1	(4) 1.6
Type C		(90) 32.7	(2) 0.8
	**Total**	**(275) 100.0**	**(247) 100.0**

*p-value<0.001.

### Distribution of schistosomiasis indicators in the re-evaluated population (1981–2005)

The school-children were used as indicators of the current situation of schistosomiasis mansoni in 2005. Thus, the prevalence rate estimated in 759 school-children was 1% for *S. mansoni*, 1.7% for hookworms, and 0.4% for *Ascaris lumbricoides*.

Table 2 shows the data related to the distribution of indicators for schistosomiasis mansoni in the re-evaluated group. The infection rate decreased dramatically (from 70.4% in 1981 to 1.7% in 2005). Infection rate in the age of group (30–40 years) in 1981 was 69.2% in 130 persons; in 2005 it was 4.1% in 98 persons. The geometric average of eggs per gram of feces was 334 epg and 172 epg, in 1981 and 2005, respectively. The patients presenting more than 500 epg in 1981 were 36.6%, whereas only one case could be detected in 2005.

**Table 2:** Distribution of schistosomiasis indicators in patients living in Comercinho/MG, Brazil (1981–2005).

VARIABLES		1981(N)%	2005(N)%
**1. Patients with stool examination**			
Yes		(1328) 90.2	(358) 75.4
No		(146) 9.8	(117) 24.6
	**Total**	**(1474) 100.0**	**(475) 100.0**
			
**2. Patients with stool examination** *			
(infection rate)		(936) 70.4	(6) 1.7
	**Total**	**(1329) 100.0**	**(358) 100.0**
			
**3. Egg countings (epg)** ***			
1–500		(565) 60.4	(5) 83.3
≥500		(371) 36.6	(1) 16.7
	**Total**	**(936) 100.0**	**(6) 100.0**
			
**4. Geometric average (epg)**		**(334)**	**(172)**

*p-value<0.001.

***It was not possible to perform statistical analysis due to the insufficient number of patients(epg) number of eggs per gram of feces.

### Data related to clinical re-evaluation and contact with natural waters of the local population in Comercinho/MG, Brazil (1981–2005)

Table 3 shows the results obtained with clinical evaluation in the studied years. The clinical form Type I was detected in 67.9% and 95.2% of the patients in 1981 and 2005, respectively. The clinical forms Type II and III were observed in 25% and 3.5%, and 6.8% and 1.3%, in 1981–2005, respectively. Among the signs and symptoms evaluated, abdominal pain and diarrhoea were the most reported at both study periods. Blood in the stools was present in 50% of the studied population in 1981, but no similar case was reported in 2005.Table 4 shows the results on contact with natural waters in Comercinho. In 1981, the daily contact with natural waters was reported by 62% of the population, whereas in 2005 by just 25%. Also, a marked decrease related to the use of natural waters was detected, when the bi-weekly or less frequency was considered (51.9% in 2005, and 13.7% in 1981). As far as the reasons for contact with natural waters were concerned, in 1981, 21% and 3.7% were due to leisure and professional activities, and in 2005 these activities were reported by 27.2% and 44.4% of the population.

**Table 3:** Comparative data of the clinical re-evaluation in Comercinho/MG, Brazil (1981–2005).

VARIABLES		1981(N)%	2005(N)%
			
**1. Clinical form** *			
**Type I**		(585) 67.9	(220) 95.2
**Type II**		(219) 25.3	(8) 3.5
**Type III**		(59) 6.8	(3) 1.3
	**Total**	**(863) 100.0**	**(231) 100.0**
**2. Signals and symptoms in Positive patients** ***			
Abdominal pain		(527) 59.7	(3) 60.0
Diarrhoea		(532) 70.4	(0) 0.0
Blood in the feces		(442) 100.0	(0) 0.0
Melena		(4) 0.5	(1) 20.0
Hematemesis		(1) 0.1	(0) 0.0
Ascites		(1) 0.1	(0) 0.0
Asymptomatic		(216) 24.5	(2) 40.0
	**Total**	**(863) -**	**(5) -**

*p-value<0.001.

***It was not possible to perform statistical analysis due to the insufficient number of patients in the sample obtained.

**Table 4:** Report on contact with natural waters in Comercinho/MG, Brazil (1981–2005).

VARIABLES		1981(N)%	2005(N)%
**1. Frequency of contact**			
Daily		(631) 62.0	(13) 25.0
Weekly		(247) 24.3	(12) 23.1
Biweekly or less		(139) 13.7	(27) 51.9
	**Total**	**(1017) 100.0**	**(52) 100.0**
**2. Reason for contact**			
Washing clothes		(209) 17.6	(8) 9.9
Fetching water		(437) 25.7	(11) 13.6
Bathing		(542) 32.0	(4) 4.9
Leisure		(356) 21.0	(22) 27.2
Professional activities		(63) 3.7	(36) 44.4
	**Total**	**(1329) 100.0**	**(358) 100.0**

## Discussion

In the last two decades, studies performed in Brazil [5], [6], [9], [10], [12], [13], [17]–[19], [32], and in other countries [20]–[27], have described the distribution of infection, reasons and frequency of contact with natural waters, as well as other parameters related to schistosomiasis mansoni.

In the present study, we studied a population from about 1,400 individuals that participated in a survey carried out in 1981, comparing the results obtained with the current ones. Nevertheless, only 358 patients were re-examined, the other ones could not be observed, since some of them moved from the town or refused to participate.

Comercinho/MG, Brazil, was considered as a hyperendemic area in 1981 (70.4%), but turned into a low endemic area (1.7%) in 2005. Administration of various treatments and quality of intervention measures produced an appreciable decrease in the prevalence of the disease (97.6%). The geometric average of the number of eggs per gram of feces obtained was 172 epg, lower than that reported by Costa in 1983 [12] (334 epg), or a little higher than the average found by Cury in 1991 [13] (105 epg), both of them also in Comercinho. It is note worthy that this actual average was obtained taking into account only 6 cases related to *S. mansoni* eggs discharged in the feces. Various studies in different regions demonstrated that the intensity of infection varies very much, and that reinfection after treatments in endemic areas show a lower number of eggs in the feces, when compared to the number detected pre-treatment [28], [29]. The rate of splenomegaly was 1.3% in adults in 2005, lower than that detected at the beginning of the project (6.8%). This fact may be connected with various treatments administered to the population along of 25 years. In fact, according to Kloetzel (1967) and Bina (1977) [28], [30], after specific treatment for schistosomiasis, even when reinfection occurs, it can be observed that the splenomegaly rate decreases significantly, and no new cases among the treated patients could be found. In Comercinho, no new case of hepatosplenomegaly could be detected after more than two decades of surveillance.

The reasons and frequency for natural water contact frequently occurs in association with the socioeconomic standard of the population living in endemic areas, and depend on their needs and cultural habits. In Comercinho in 2005, the main reasons for contact with natural waters pointed to professional activities, such as: watering vegetable-garden or farming, removing sand, crossing a brook, etc. (44.4%). The decrease of the related daily contact may be directly connected with the increase in the number of households with water supply. However, it was not possible to correlate directly the contact with natural waters to infection with *S. mansoni*, in the last survey, since only three positive patients mentioned contact with natural waters, with a biweekly frequency. Costa et al. (1987) [33] reported that the main risk factors responsible for splenomegaly in Comercinho were: absence of piped water, daily contact with natural waters and unskilled workers. Scott et al. (2003) [24] showed that many aspects, such as frequency, duration or time of contact, have influence on the infection rate.

Certainly, the supply of safe water at town level diminished the incidence of schistosomiasis, since the existence of piped water in the housings reduced considerably the frequency and duration of contact with natural waters.

In 231 patients clinically examined in 2005, 95.2% presented intestinal clinical form of the disease, 3.5% showed hepatointestinal and 1.3% hepato-splenic forms, whereas at the beginning of the study, the percentages were 67.9%, 25.3% and 6.8%, respectively. The reversal of hepatomegaly and splenomegaly was deemed as significant.

The importance of treatment and provision of sanitation for decrease of prevalence and morbidity control was previously emphasized. In Capitão Andrade, a small town in the State of Minas Gerais, Brazil, Conceição & Pereira (2002) [9] noticed that over a 21-year-period, from 1973 to 1994, the prevalence decreased (60.8% in 1973; 32.2% in 1984, and 27.3% in 1994), whereas the evolution profile of the clinical forms was found to be satisfactory (unaltered in 76.7%, clinical progression in 8.4% and regression in 14.9%). The reduction of both prevalence and severity of *S. mansoni* infection were ascribed to the treatment with oxamniquine administered in all infected individuals in 1984, as well as to provision of piped water in the housings. In 2003, those authors re-evaluated the area, and observed that the prevalence has also decreased (19.4%) in relation to the preceding years, as well as the hepato-splenomegaly (5.8% in 1973, 2.8% in 1984, 2.3% in 1994 and 1.3% in 2003). They observed that in spite of the significant reduction in the prevalence of infection without treatment at the initial phase (1973–1974), followed by a specific treatment with oxamniquine in 1984–1994, the rate of the severe forms and prevalence remained very high throughout the period 1994–2003. During this time, people continued to receive treatment, but there were no improvements related to either basic sanitation or potable water supply, only sanitary education was strengthen. Thus, these facts led to the supposition that the high prevalence and severity of the clinical forms may have occurred due to reinfection [10].

In our laboratory, a study was devised to be carried out in Ravena, a district of Sabará, State of Minas Gerais, Brazil, in 1980. Initially, the prevalence of schistosomiasis in Ravena was 36.7%, with an infection intensity of 229 epg (geometric average related to positive individuals). No cases of hepato-splenic form could be detected. A specific treatment with oxamniquine in large scale was provided (every four years, three treatments) to patients discharging eggs in the feces. In 1992, the local population was re-examined. When the study was initiated, 90% of the housings received safe water supply. The number of housings with an appropriate waste disposal also increased (from 17% to 36%). In 1992, the prevalence in the population decreased to 11.5%, and the average of eggs was 60.3 [34], [35]. Recently, the same area was re-examined, *i.e.*, 27 years after the first clinical-epidemiological survey [11]. In this last survey, the prevalence was 2.5%, with an average of 21 epg. In the age group of 0–14 years old the positivity rate was 0.75, whereas in 1980 this rate was 11.6%. Besides, 95% of the housings disposed of safe water supply, and more than 80% had appropriate waste disposal either by means of sewerage system, flush toilets or cess-pits. From 1990 onwards, the population was treated by a physician at the local health center, based on the results of stool examinations and by spontaneous plea. The living standard related to water contact in Ravena was modified throughout the years, since the majority of the population is no more in the habit of using natural contaminated water.

In the last survey in Comercinho, 96% of the houses visited disposed of safe water supply by means of the public system, 97.6% had flush toilets or cess-pits for waste disposal, and 97.6% of the housings were classified as being of better quality (Type A).

The composition of a schistosomiasis control program varies according to two approaches: 1. Control of morbidity, aiming at reducing the number of severe form of the disease; 2. Control of transmission, by interrupting the evolutive cycle of the parasite. In the first case, the control of morbidity is specially undertaken by using chemotherapy, whereas the control of transmission requires treatment, safe water supply and appropriate waste disposal, environmental sanitation, and health education [2], [31].

Currently, in Comercinho, low prevalence rate regarding the population in general and in previously treated individuals, low frequency of cases with hepatosplenic form, have clearly proved that control measures in association can led to interruption or significant decrease of transmission. At least, this clearly happens in Ravena and Comercinho.

Finally, due to the effectiveness of the measures above mentioned, it is quite clear that the Brazilian Government should adopted the association of control measures mentioned in this study in order to attain schistosomiasis transmission control in the country.

## Supporting Information

Checklist S1 Strobe checklist
(0.09 MB DOC)
